# Supplementary material for: The interactome of intact mitochondria by cross-linking mass spectrometry provides evidence for coexisting respiratory supercomplexes
Source: Mol Cell Proteomics. 2017 Dec 8;17(2):216–32. doi: 10.1074/mcp.RA117.000470 (PMC5795388; doi:10.1074/mcp.RA117.000470)
Supplement: Supplemental Data [file supp_RA117.000470_133922_0_supp_23978_fzffwf.zip › spectra_annotation/mito_DR_spectra_annotation/334-1-6-1-6-1.pdf]

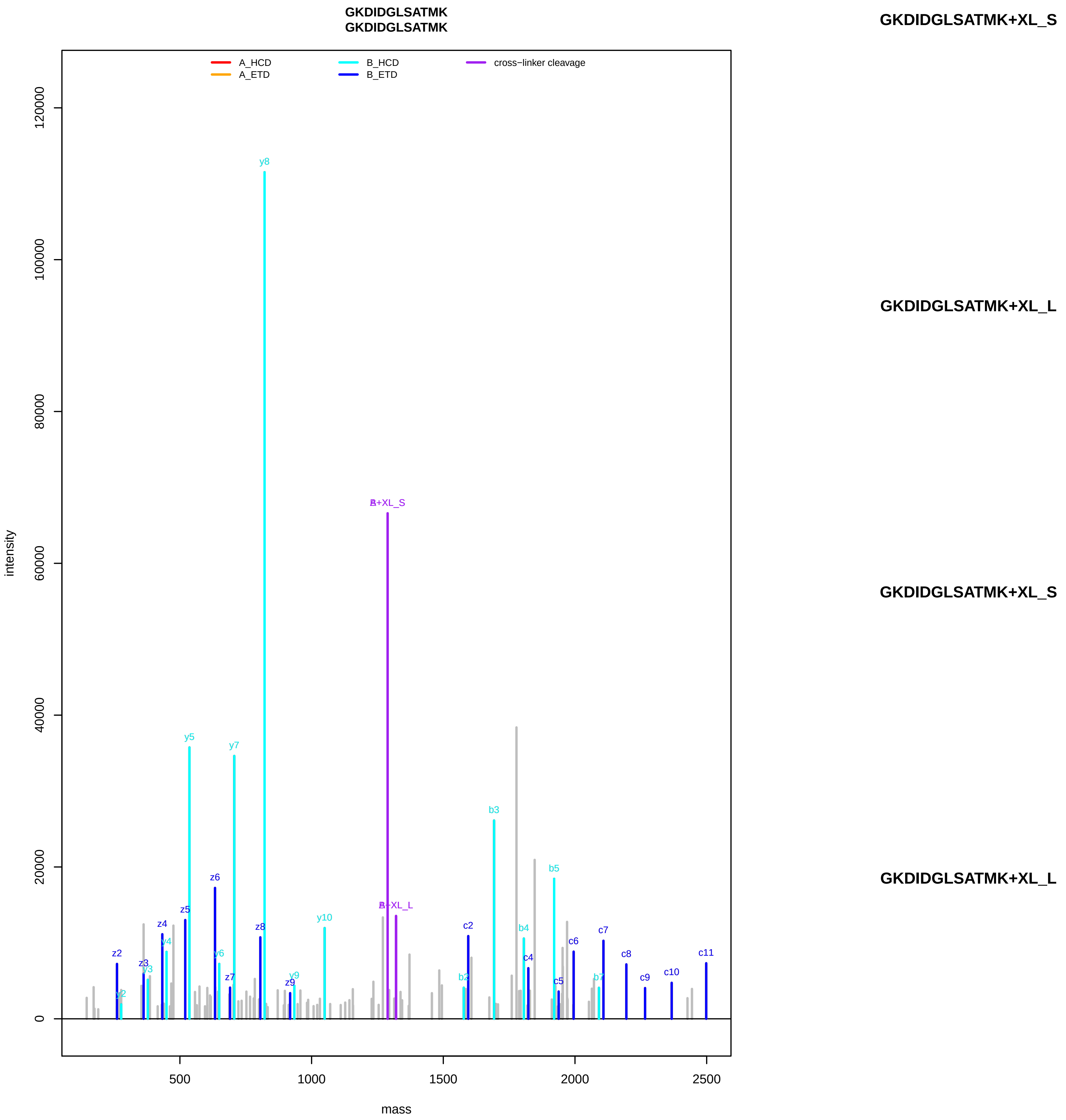

GKDIDGLSATMK
GKDIDGLSATMK
A_HCD
A_ETD
B_HCD
B_ETD
cross−linker cleavage
intensity
mass
y8
B+XL_S
B+XL_L
y5
y7
b3
b5
z6
z5
z4
z8
y10
c2
b4
c7
c6
c4
c8
c11
c10
c9
c5
b7
b2
z2
z3
y3
y4
y6
z7
z9
y9
y2
0
20000
40000
60000
80000
100000
120000
500
1000
1500
2000
2500
GKDIDGLSATMK+XL_S
GKDIDGLSATMK+XL_L
GKDIDGLSATMK+XL_S
GKDIDGLSATMK+XL_L
